# Ameliorative Effects of *Humulus japonicus* Extract and Polysaccharide-Rich Extract of *Phragmites rhizoma* in Rats with Gastrointestinal Dysfunctions Induced by Water Avoidance Stress

**DOI:** 10.1155/2022/9993743

**Published:** 2022-01-21

**Authors:** Wynn Thein, Won Seok Choi, Wah Wah Po, Tin Myo Khing, Ji Hoon Jeong, Uy Dong Sohn

**Affiliations:** ^1^Department of Pharmacology, College of Pharmacy, Chung-Ang University, Seoul 06974, Republic of Korea; ^2^Department of Pharmacology, College of Medicine, Chung-Ang University, Seoul 06974, Republic of Korea

## Abstract

Chronic stress can cause the gastrointestinal disorders characterized by an altered bowel movement and abdominal pain. Studies have shown that *Humulus japonicus* extract (HJE) has anti-inflammatory and antidiarrheal effects, and *Phragmites rhizoma* extract (PEP) has antioxidative and antistress effects. The present study aimed to investigate the possible effects of HJE and PEP in rat models with stress-induced gastrointestinal dysfunctions. The rats were exposed to water avoidance stress (WAS, 1 h/day) for 10 days to induce gastrointestinal disorders. We found that WAS significantly increased fecal pellet output during 1 h stress, gastric emptying, colonic contractility, and permeability compared to the normal rats. Pretreatment with HJE and PEP (0.25 and 0.5 mL/kg, both administered separately) improved the increased gastric emptying and colonic contractility induced by electrical field stimulation, acetylcholine, and serotonin and also alleviated the increased colonic permeability. HJE and PEP also increased the claudin-1 and occludin expressions, reduced by WAS. WAS increased the concentration of TNF-*α* and TBARS and reduced FRAP. HJE and PEP recovered these effects. HJE and PEP improved the gastrointestinal disorders induced by WAS by upregulating the tight junction protein, possibly acting on cholinergic and serotonergic receptors to abolish the colonic hypercontractility and hyperpermeability and degradation of inflammatory cytokines via an antioxidant effect.

## 1. Introduction

Irritable bowel syndrome (IBS) is a common functional gastrointestinal (GI) disorder characterized by abdominal pain or discomfort, stool irregularities, and bloating [1]. IBS is a multifactorial disease; the underlying causes are still complex, and the true pathophysiology is far from being understood. Psychological pressure is an important cause of IBS. Chronic water avoidance stress (WAS) was considered as one of the most effective psychological stressors in rodents with IBS symptoms. WAS provoked colonic hyperalgesia and increased colonic motility and gut hyperpermeability [2, 3].

In GI homeostasis, the intestinal epithelial barrier is the first line of defense against numerous adverse factors, including toxins, certain antigens, and pathogenic microorganisms. Tight junctions (TJs) regulate the selective/semipermeable transportation of paracellular solutes and intercellular signaling responses. TJs are a series of protein complexes (claudins, occludin [OCLN], and junctional adhesion molecules) that perform dynamic functions [4, 5]. Changes in the structure and functions of the TJ protein complexes are associated with epithelial barrier disruption. The disruption allows the entry of inflammatory mediators to promote low-grade inflammation and visceral hypersensitivity [6]. In response to electrical field stimulation (EFS) of the colon, there is simultaneous stimulation of all neurons within the myenteric plexus, including excitatory and inhibitory components, sympathetic and parasympathetic nerves, and cholinergic, nonadrenergic, and noncholinergic neurotransmitters [7]. The neurotransmitter acetylcholine (ACh) acts on the smooth muscle muscarinic receptors and is a determinant of colonic contractility [8]. Moreover, serotonin or 5-hydroxytryptamine (5-HT) is an important neurotransmitter and regulates GI motility. The myeloperoxidase activity, oxidative stress, IL-6, IL-1, activated neutrophils, and eosinophils play crucial roles in inflammatory reaction in the pathogenesis of IBS [9–11].

Medicinal plants have been used to treat and prevent various diseases. *Humulus japonicus* is a plant belonging to the family Cannabaceae. *Humulus japonicus* extract (HJE) had been previously reported to possess potent antioxidative, antimycobacterial, anticancer, antibacterial, antimutagenic, and anti-inflammatory properties [12]. It has been documented that HJE ameliorated hydrogen peroxide-induced oxidative stress in mouse adipocytes [13]. The plant has been used as traditional medicine in Korea to treat dysentery, chronic colitis, pulmonary tuberculosis, and hypertension [14]. The polysaccharide-rich extract of *Phragmites rhizoma* (PEP), another medicinal plant belonging to the family of Poaceae, possesses antioxidative, free radical scavenging properties, and anti-inflammatory properties in atopic dermatitis effects [15, 16]. In our previous studies, PEP reduced the bleeding and ulcer formation of stomach in forced swimming rats [17]. The dried root of *Phragmites rhizoma* has been used to alleviate fever and vomiting in traditional Korean medicine. It is also registered in Korean Herbal Pharmacopoeia and Chinese Pharmacopoeia [18]. However, the effects of HJE and PEP on GI disorders induced by WAS have not yet been elucidated.

Taking into consideration the biological activities of HJE and PEP on the GI tract, the present study aimed to explore the effects of HJE and PEP in rat models with gastrointestinal disorders and the underlying mechanism of HJE PEP in GI disorders from two aspects: colonic permeability and colonic contractility. Furthermore, the current study targeted the evaluation of the anti-inflammatory and antioxidant effects on HJE- and PEP-treated rat models with GI disorders.

## 2. Materials and Methods

### 2.1. Animals

**Figure 1:**
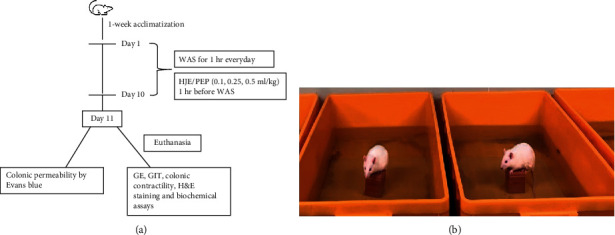
Schematic representation of experimental protocols. General experimental protocol (a) and gastrointestinal disorders rat models by water avoidance stress (WAS) (b). WAS test was done for 1 h every day for 10 consecutive days.

All experiments were performed on adult male Wistar rats (weighing 250–300 g) obtained from Daehan Biolink (DBL, Seoul, Korea). Animals were housed in plastic cages, with two rats per cage at room temperature and 65%–70% relative humidity. Animals were housed on a 12 h light/12 h dark cycle, with free access to water and food. The animals were acclimatized in the animal center for 1 week before the experiments. They were then divided into 9 groups: normal, IBS, loperamide-treated (5 mg/kg), HJE-treated (0.1, 0.25, and 0.5 mL/kg), and PEP-treated (0.1, 0.25, and 0.5 mL/kg) groups. The drugs and medicinal plant extracts were given 1 h before WAS protocol. The rats were fasted 24 h with tap water for gastric emptying (GE) and gastrointestinal transit (GIT) experiments. All rat experiments were conducted in accordance with the Guide for the Care and Use of Laboratory Animals of the National Institutes of Health [19] and approved by the Institutional Animal Care Use Committee of the Chung-Ang University of Korea (approval number: IACUC 2019-00111). The schematic representation of the experimental protocol was provided in Figure 1(a).

### 2.2. Reagents

Aqueous extracts HJE and PEP were generously provided by Professor Ji Hoon Jeong (Department of Pharmacology, College of Medicine, Chung-Ang University) on June 25, 2019. Tissues for colonic contractility were maintained in Krebs buffer [NaCl, 116.6 mM; NaHCO_3_, 21.9 mM; NaH_2_PO_4_, 1.2 mM; KCl, 3.4 mM; MgCl_2_, 1.2 mM; glucose, 5.4 mM; and CaCl_2_, 2.5 mM]. OCLN, claudin-1, and *β*-actin were obtained from Santa Cruz Biotechnology (Santa Cruz, CA, USA). Loperamide, Evans blue, 10% buffered formalin, reagent alcohol, *N*-acetyl cysteine, carboxymethylcellulose, phenol red, trichloroacetic acid, acetylcholine, serotonin, aprotinin, leupeptin, phosphate inhibitor cocktail-3, thiobarbituric acid, n-butanol, malonaldehyde, tripyridyl triazine (TPTZ), and ferrous sulfate were purchased from Sigma (St. Louis, MO, USA).

### 2.3. Preparation of HJE and PEP

The HJE was prepared according to the previously described work [20]. The lyophilized water extract was prepared from dried HJ that was obtained from Woori Oriental Medicine Materials and authenticated by Dr. Yuan Lu Sun of Solvit P&F (Seoul, Republic of Korea). 650 g of dried HJ was mixed with 15 L distilled water and boiled for 4 h at 100°C in duplicate. The extract was filtered and the filtrate was evaporated up to 15.6% and finally lyophilized using freeze-drying lyophilizer (Labconco; Freezone 1 L) at 5 mmHg and −50°C. The lyophilized powder was stored at −30°C pending analysis. Ultra performance liquid chromatography and high-resolution mass spectrometer analysis were performed with a Dionex 3000 RSLC and Thermo Scientific Q Exactive^TM^ (Hybrid Quadrupole-Orbitrap Mass Spectrometer, Bremen, Germany). The chromatographic separation was carried out on the ACQUITY BEH C18 column (2.1 × 100 mm, 1.7 *μ*m). The mobile phase consisted of 0.1% formic acid in water, and acetonitrile was flowed at a rate of 0.35 mL/min; 10 *μ*L of HJ (10 mg/mL) was injected. Compounds were identified using standards of vitexin, luteolin, luteolin 7-O-*β*-d-glucoside, apigenin, and chlorogenic acid. Negative ion mode electrospray ionization mass spectrometry was used for the detection of all analytes. An ion spray voltage of 3.5 kV and an S-lens level of 50 were used. Capillary and heater temperatures were set at 350°C and 100°C, respectively.

The PEP was prepared as reported previously [17]. Briefly, the sliced and dried raw materials of *Phragmites rhizoma* were blanched at 100°C for 4 h (43.3 g/L of water) and allowed to cool down to room temperature. After cooling, the supernatant was applied to a Sephadex G-150 (2.7 × 72 cm) column, and bound materials were eluted with a linear gradient of 0.1 M NaCl. The fractions containing carbohydrates were pooled and precipitated thrice with ethanol. The resulting polysaccharide extract was freeze-dried for preparing the PEP powder. The concentration of carbohydrates in the fraction was determined using the phenol-sulfuric acid methods [21].

### 2.4. Induction of Chronic Water Avoidance Stress (WAS) Test in Rats

GI disorders were induced by WAS in rats. Repeated WAS has both excellent face and construct validity for IBS [2]. Briefly, the test apparatus included a plastic tank (45 cm length × 25 cm width × 25 cm height) with a block (10 × 8 × 8 cm) placed in the center of the plastic floor. The tank was filled with fresh water (25°C) to 1 cm from the peak of the block. The rats were positioned on the block for a duration of 1 h daily for 10 consecutive days corresponding to the chronic stress protocol. Control animals were individually placed in the same plastic cage, which was not filled with water. Representative photographs of the WAS tests are seen in Figure 1(b). The number of fecal pellets was recorded during the WAS session.

### 2.5. Evaluation of HJE and PEP on the GI Motor Functions

#### 2.5.1. Measurement of GE

GE was measured by the previously described method [22]. Briefly, after 10 days of WAS, the rats were fasted for 24 h before the experiment. Then, 15 min before euthanasia of the rats, 1 mL of phenol red solution (1 L of distilled water, 5 g of carboxymethylcellulose, and 5 g of phenol red) was administered orally. The rats were euthanized by CO_2_ asphyxiation. The stomach was clamped from the pylorus to cardia and removed. The stomach was then homogenized in 5 mL of 0.1 N NaOH solution. This homogenate was precipitated with 0.5 mL of trichloroacetic acid (TCA, 20% w/v) and centrifuged at 5000*g*, 4°C for 10 min. Afterwards, 0.1 mL supernatant was mixed with 0.1 mL of 0.5 N NaOH aqueous solution. The absorbance at a wavelength of 590 nm was measured with the FlexStation® 3 devices (Molecular Devices Co., San Jose, CA, USA). The rate of GE in terms of GE (%) was calculated using the following formula:

(1) $$\begin{array}{c} \text{Gastric Emptying }\left(\text{\%}\right)=\left[1-\frac{{\text{Absorbance}}_{590\text{ nm}}\text{ of test stomach}}{{\text{Absorbance}}_{590\text{ nm}}\text{ of }0\text{ time control stomach}}\right]\times 100. \end{array}$$

#### 2.5.2. Measurement of GIT

GIT was measured with a modified method of GE. The entire small intestine was removed from the same rat of GE experiments and then divided into 10 segments. Each segment was homogenized in 0.7 mL of 0.1 N NaOH aqueous solution. The homogenate was precipitated with 0.2 mL TCA (20% w/v) and centrifuged. In a 96-well plate, 0.1 mL supernatant was added to 0.1 mL of 0.5 N NaOH aqueous solution. The absorbance at a wavelength of 590 nm was determined. The GIT as a mean geometric center (MGC) was calculated by using the following formula:

(2) $$\begin{array}{c} \text{Mean Geometric Center }\left(\text{No}.\right)=\frac{\sum_{k=1}^{10}a_{k}{}^{*}b_{k}}{\sum_{k=1}^{10}a_{k}}, \end{array}$$

where *a*_*k*_ is absorbance at 590 nm of each segment and *b*_k_ is segment number (1, 2,…, 10).

#### 2.5.3. Measurement of Colonic Contractility

On day 11, the rat was euthanized by CO_2_ asphyxiation. The colonic contractility was measured according to the previous work [23]. The colon tissue was collected and cut into two smaller strips (3 mm width × 7 mm length), tied with silk ligatures, and mounted in a 1 mL muscle chamber. The end of the silk was connected to the force transducer (FT03, Grass Instruments Co., Quincy, MA, USA). Changes in isometric force were recorded on a polygraph (Model 79, Grass Instruments Co.). The colon strips were stretched to 1 g and were kept at equilibrium for 90 min in Krebs buffer containing organ chambers with continuous oxygen supply at 37°C. Ten minutes after passive tension, the muscle reached the baseline.

EFS was performed as follows: pulse trains of 40 V in amplitude and 10 s in train duration and pulse duration of 1 ms at frequencies of 1, 2, 4, and 6 Hz by using a stimulator (Model S88; Grass Instruments Co.). In addition, ACh (10^−4^ M) and 5-HT (10^−4^ M) were added to separate organ baths to stimulate the colonic contraction. The responses of the colonic strips to EFS, ACh, and 5-HT were calculated by recording the changes from the baselines to the maximum responses.

### 2.6. Evaluation of HJE and PEP on Colonic Inflammation as Well as Colonic Permeability and Oxidative Stress

#### 2.6.1. Measurement of Colonic Permeability

Colonic permeability was measured as reported previously [24]. After 10 days of WAS, rats from each group were used to determine the colonic permeability using Evans blue. The rats were anesthetized by an intraperitoneal injection of tiletamine/zolazepam (30 mg/kg, Zoletil, Virbac, France). Then, cecal ligation was performed after laparotomy. An open-tipped catheter was inserted into the proximal colon through the small puncture made by a needle at 1 cm distal from the ligation. Phosphate-buffered saline (PBS, 37°C) was used to gently flush the colon through the catheter, removing the stools. The colon was again ligated at approximately 4 cm from the first ligation, and 1 mL of 1.5% Evans blue in PBS was injected into the ligated colon segment through the catheter. After 15 min, the rat was euthanized, and the colon was excised, washed with PBS and 1 mL of 6 mM *N*-acetyl cysteine, opened, and then placed n 2 mL of *N*,*N*-dimethylformamide for 12 h to extract the Evans blue from the colon tissue. Standard Evans blue concentrations ranging from 0 to 128 *μ*g/mL were prepared. The absorbance of the supernatant was measured using a spectrophotometer at 610 nm to determine permeability. The concentration of Evans blue was calculated in form of micrograms per gram of tissue from the absorbance against the standard curve.

#### 2.6.2. Protein Sample Preparation and Western Blotting

To measure the TJ proteins (occludin, claudin-1), the protein sample was prepared as previously described [25]. Generally, the colon tissues were homogenized in ice-cold Pierce™ RIPA buffer (Thermo Fisher Scientific, Inc., MA, USA) containing 10 *μ*g/mL leupeptin, 10 *μ*g/mL aprotinin, and 10 *μ*L/mL phosphatase inhibitor cocktail-3. The homogenates were sonicated (30 s twice, 8 cycles, power 20%) and then kept at 4°C under constant agitation for 2 h. The samples were centrifuged at 12,000*g*, 4°C for 20 min. The supernatant was used to determine the protein concentration using Pierce BCA (bicinchoninic acid) protein assay kit (Thermo scientific, Rockford, IL, USA) and western blot analysis.

Immunoblotting analysis of OCLN, claudin-1, and *β*-actin was performed. The lysates were subjected to electrophoresis on sodium dodecyl sulfate-polyacrylamide gel electrophoresis (SDS-PAGE) and transferred to nitrocellulose membranes using the Power Pac (Bio-Rad, Melville, NY, USA). Following incubation with the primary antibodies (1 : 1000) at 4°C overnight, the membranes were incubated with horseradish peroxidase- (HRP-) conjugated secondary antibodies at room temperature for 1 h. Protein bands were examined using an enhanced chemiluminescence reagent (ELPIS-Biotech., Inc, Daejeon, Republic of Korea) and exposed to X-ray photographic films (Thermo Scientific, Inc.) in a dark room. ImageJ (National Institutes of Health, Bethesda, MD, USA) was used to analyze the image data.

#### 2.6.3. Histological Evaluation

To evaluate the histological measurement of colonic permeability, the colon was obtained after 10 days of WAS, fixed in 10% buffered formalin, and dehydrated in a series of increasing concentrations of ethanol. Tissues were embedded in paraffin and cut into slices of 5 *μ*m in thickness. Structural changes in the colon were determined by staining with hematoxylin and eosin (H&E). Images of the stained sections were recorded with a Leica DM IL LED microscope (Leica Microsystems, Wetzlar, Germany). Photomicrographs were taken at ×100 magnification.

#### 2.6.4. Determination of TNF-*α* Concentration in the Colon Tissue

The colon tissues were homogenized and centrifuged at 10,000*g*, 4°C for 15 min, and then the supernatants were transferred to new Eppendorf tube and kept at −80°C before analysis. The protein content of the supernatant was quantified using the BCA assay. The concentrations of TNF-*α* in the colon were determined by using an ELISA kit for rats (E0764Ra, Bioassay Technology laboratory, Shanghai, China) according to the manufacturer's instructions.

#### 2.6.5. Ferric Reducing Antioxidant Power (FRAP) Assay

The total antioxidant capacity of the colon tissue homogenate was determined by measuring its ability to reduce Fe^3+^ to Fe^2+^ by the FRAP test [26]. The FRAP assay measures the change in absorbance at 593 nm due to the reduction of Fe^3+^ to a blue-colored Fe^2+^–tripyridyltriazine (TPTZ) compound by electron-donating antioxidants. The FRAP reagent consists of 300 mM acetate buffer pH = 3.6, 10 mM TPTZ in 40 mM HCl, and 20 mM FeCl_3_·6H_2_O in the ratio of 10 : 1 : 1. Briefly, 10 *μ*L of tissue homogenate was added to 300 *μ*L FRAP reagent (freshly prepared and prewarmed at 37°C) and incubated at 37°C for 10 min. The absorbance of the blue-colored complex was measured against a reagent blank (300 *μ*L FRAP reagent + 10 *μ*L distilled water) at 593 nm. Standard solutions in the range of 100 to 1000 mM Fe^2+^ were prepared from ferrous sulfate (FeSO_4_·7H_2_O) in water. The FRAP value was expressed as millimoles per microgram of protein.

#### 2.6.6. Thiobarbituric Acid-Reactive Substance (TBARS) Assay

Lipid peroxidation was determined according to the previously described method [27]. Briefly, 0.3 mL of the supernatant was added to the 0.9 mL of 8% TCA, and the mixture was vortexed. Following centrifugation at 10,000*g*, 4°C, for 5 min, 0.25 mL of 1% TBA was added to 1 mL of the supernatant and the solution was then boiled at 100°C for 30 min. The tubes were cooled down immediately with tap water, then mixed, and vortexed with 2 mL of *n*-butanol for 90 s. After centrifugation at 3000*g*, 4°C, for 5 min, the butanol phase was used for the TBARS assay, wherein the formation of TBARS was measured by reading the absorbance at 532 nm using a spectrophotometer. Malonaldehyde bis (dimethyl acetal) was used as the standard. The absorbance was normalized with protein concentration.

#### 2.6.7. Statistical Analysis

All data were expressed as the mean ± standard error of the mean (SEM) of at least six independent experiments. Statistical differences among the groups were analyzed by one-way ANOVA and two-way ANOVA using GraphPad Prism 8 (GraphPad, San Diego, USA) and the figures were obtained by using Excel (Microsoft, Redmond, WA, USA). Overall, a *P* value of 0.05 or less was considered statistically significant.

## 3. Results

### 3.1. Effect of HJE and PEP on the GI Motor Functions

HJE and PEP reduced the frequency of defecation in WAS rats.

**Figure 2:**
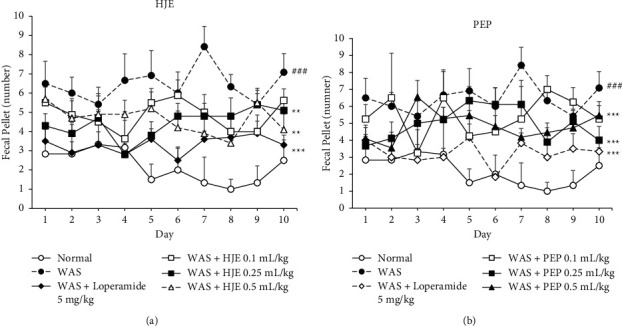
Effect of HJE and PEP on defecation in IBS rats. The number of fecal pellet outputs was recorded during 1 h of water avoidance stress in normal, WAS, loperamide (5 mg/kg), HJE-treated (0.1, 0.25, and 0.5 mL/kg) (a), and PEP-treated (0.1, 0.25, and 0.5 mL/kg) groups (b). ^###^*P* < 0.001 versus normal group. ^*∗∗*^*P* < 0.01 and ^*∗∗∗*^*P* < 0.001 versus WAS group (overall effect for each treatment) by two-way ANOVA. Data are expressed as mean ± SEM (*n* = 8).

The number of fecal granules was recorded within 1 h of WAS. There was a significant increase in the number of fecal granules collected from the WAS rats compared to normal rats (Figures 2(a) and 2(b)). Loperamide (5 mg/kg) treatment decreased the defecation in WAS rats. After treatment with HJE (0.25 and 0.5 mL/kg) and PEP (0.25 and 0.5 mL/kg) for 10 days at 1 h before WAS, the number of fecal granules collected from these rats was significantly lower than that from the WAS group. The significant differences are based on the overall effect of each treatment.

### 3.2. Improvement of GE in the Stomach by HEJ and PEP

**Figure 3:**
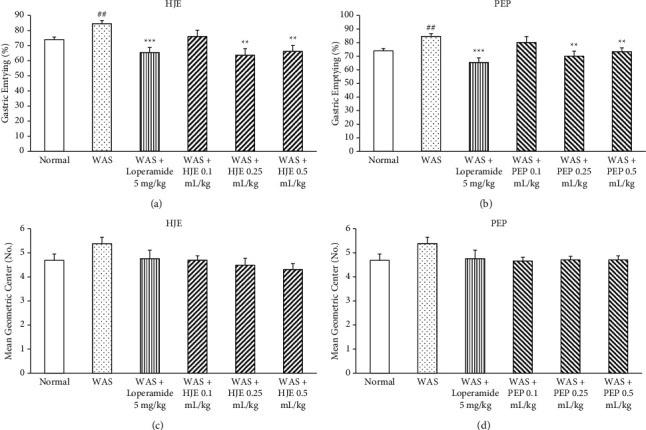
Effect of HJE and PEP on GE and GIT in WAS rats. Gastric emptying (GE) and gastrointestinal transit (GIT) values were measured from the rat stomach and small intestine by phenol red administration. The inhibition of HJE and PEP on GE (a, b). Effect of HJE and PEP on GIT (c, d). ^##^*P* < 0.01 versus normal rats. ^*∗∗*^*P* < 0.01 and ^*∗∗∗*^*P* < 0.001 versus WAS rats. Data were expressed as mean ± SEM (*n* = 8).

The rate of GE was used to measure the motility of the stomach. There was a significant increase in the GE of the WAS group compared to the normal group (Figures 3(a) and 3(b)). By contrast, loperamide treatment and HJE (0.25 and 0.5 mL/kg) treatment (*P* < 0.01) decreased the GE of the stomach significantly compared to the WAS group (Figure 3(a)). In addition, when the rats were pretreated with PEP (0.25 and 0.5 mL/kg), the increased GE in IBS rats was reversed significantly (Figure 3(b)). At 0.1 mL/kg, both HJE and PEP did not recover the increased GE in WAS rats.

### 3.3. Effect of HJE and PEP in GIT of WAS Rats

The GI motility was estimated by measuring GIT in the small intestine as MGC. WAS group did not increase the MGC compared to the normal group. There were also no significant differences between HJE-treated and PEP-treated groups and the WAS group (Figures 3(c) and 3(d)).

### 3.4. HJE and PEP Inhibited the EFS, ACh, and 5-HT-Induced Contractile Responses in WAS Rats

**Figure 4:**
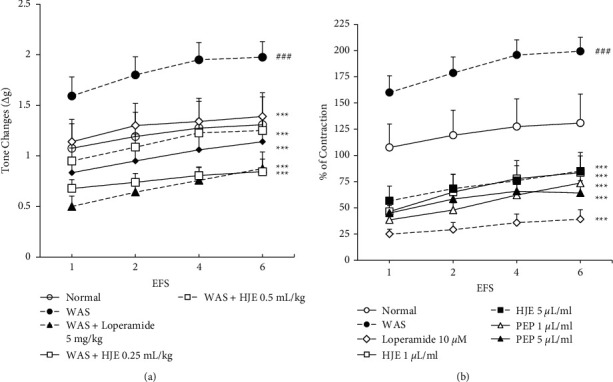
Effect of HJE and PEP on EFS-induced colonic contractility in WAS rats. The effect of 10-day pretreatment with 0.25 and 0.5 ml/kg of HJE and PEP on EFS-induced contractility in WAS rats (a). The effect of HJE and PEP (extract was added at 1 and 5 *μ*L/ml, to the tissue organ bath 15 min before EFS) on EFS-induced colonic motility in WAS models (b). ^###^*P* < 0.001 versus normal groups; ^*∗∗∗*^*P* < 0.001 versus WAS group by two-way ANOVA. Data were expressed as mean ± SEM (*n* = 8).

**Figure 5:**
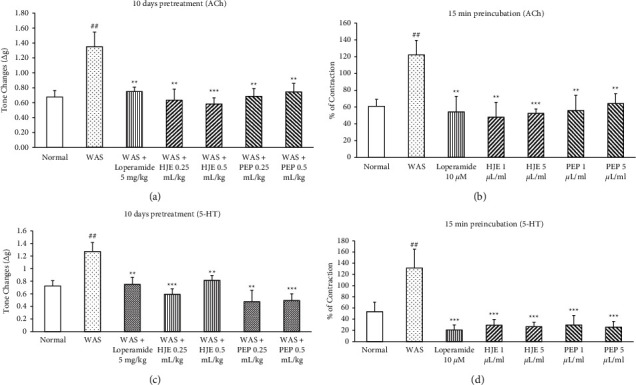
Effect of HJE and PEP on acetylcholine- and serotonin-induced colonic contractility in WAS rats. The effect of 10-day pretreatment with 5 mg/kg of loperamide and 0.25 and 0.5 ml/kg of HJE and PEP on acetylcholine-induced (a) and serotonin-induced (c) motility in WAS rats. The effect of loperamide (10 *μ*M) and HJE and PEP (1 and 5 *μ*L/ml; extract was added to the tissue organ bath 15 min before stimulation) on acetylcholine-induced (b) and serotonin-induced (d) colonic contractility in WAS rats. ^##^*P* < 0.05 versus normal groups; ^*∗∗*^*P* < 0.01 and ^*∗∗∗*^*P* < 0.001 versus WAS group. Data were expressed as means ± SEM (*n* = 8).

To establish whether cholinergic or serotonergic signaling was involved in IBS, colonic contractility was induced by EFS (1, 2, 4, and 6 Hz), ACh (10^−4^ M), and 5-HT (10^−4^ M). The contractile responses induced by EFS (Figure 4(a)), ACh (Figure 5(a)), and 5-HT (Figure 5(c)) were significantly increased in the WAS group compared to the normal group. Ten-day pretreatment with loperamide (5 mg/kg), HJE (0.25 and 0.5 mL/kg), and PEP (0.25 and 0.5 mL/kg) abolished this response significantly (Figures 4(a), 5(a), and 5(c)).

Loperamide (10 *μ*M), HJE (1 and 5 *μ*L/mL), and PEP (1 and 5 *μ*L/mL) were also preincubated with the WAS colonic smooth muscle strips 15 min before the EFS, ACh, and 5-HT stimulation in separate organ baths. A significant increase in the colonic contraction of the IBS group due to EFS, ACh, and 5-HT was observed compared to the normal group. Preincubation with loperamide, HJE, and PEP significantly blocked the increased contraction in the WAS colon (Figures 4(b), 5(b), and 5(d)).

### 3.5. Effect of HJE and PEP on Colonic Permeability and Tight Junction Proteins in WAS Rats

**Figure 6:**
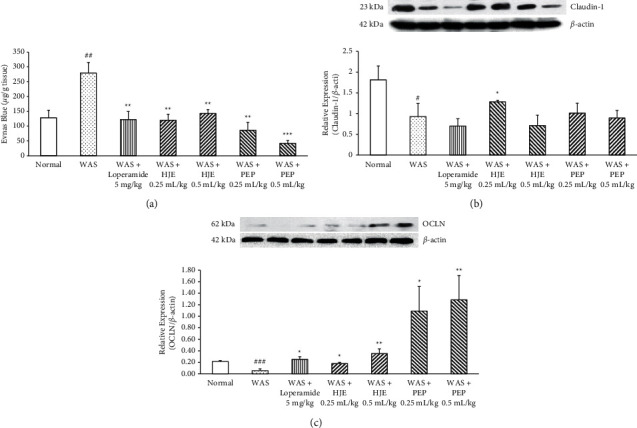
Effect of HJE and PEP on increased colonic permeability and expression of tight junction proteins in WAS rats. Effects of HJE and PEP on colonic hyperpermeability by Evans blue in WAS rats (a). All rats were exposed to WAS session except the normal rats. Data were expressed as means ± SEM (*n* = 3). Effect of HJE and PEP on the expression of claudin-1 (b) and occludin (c) protein in WAS rats. Protein samples were prepared from the colon tissues. Protein expression was determined by western blot. ^##^*P* < 0.01 and ^#^*P* < 0.05 versus normal group. ^*∗*^*P* < 0.05, ^*∗∗*^*P* < 0.01, and ^*∗∗∗*^*P* < 0.001 versus WAS group.

Colonic permeability was increased significantly in the WAS group compared to the normal group. Ten-day pretreatment with loperamide (5 mg/kg) and HJE (0.25 and 0.5 mL/kg) significantly abolished this response. Likewise, PEP (0.25 and 0.5 mL/kg) significantly decreased the colonic hyperpermeability in WAS rats (Figure 6(a)). Western blot analysis of the TJ protein claudin-1 (Figure 6(b)) revealed a significantly reduced claudin-1 content in the WAS group compared to the normal group. Pretreatment with low-dose HJE (0.25 mL/kg) markedly elevated the claudin-1 expression compared to the WAS group, in contrast to the claudin-1 expressions in the loperamide (5 mg/kg), high-dose HJE (5 mL/kg), and PEP (0.25 and 0.5 mL/kg) group, and WAS group.

Analysis of the expression of the TJ protein OCLN by western blot showed that it was significantly downregulated in WAS rats compared to the normal rats. The loperamide-treated rats as a positive control increased the expression of OCLN when compared to the WAS rats. Similarly, all HJE- and PEP-pretreated rats upregulated the OCLN expression markedly compared to the WAS rats (Figure 6(c)).

### 3.6. HJE and PEP Improved the Ulceration and Inflammation of the Colon in WAS Rats

**Figure 7:**
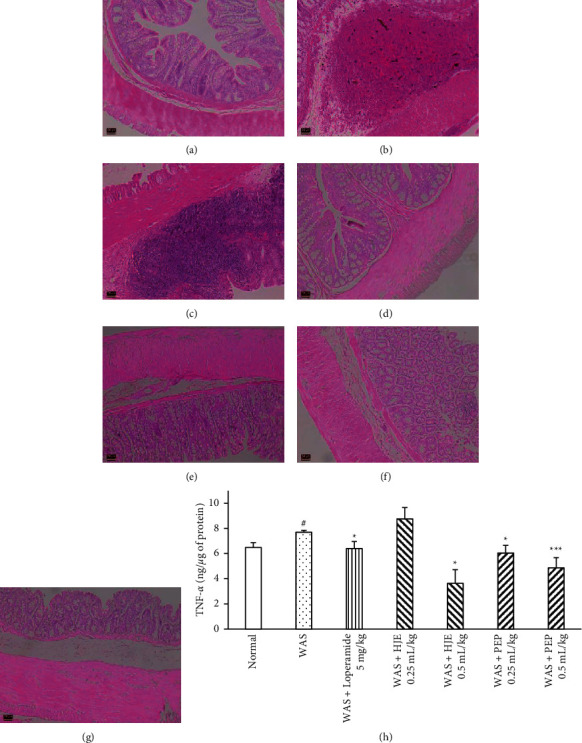
Effect of HJE and PEP on inflammation in WAS colon. Histological comparison of the WAS colon at day 11. Stained colon sections in normal group (a), WAS group (b), loperamide 5 mg/kg + WAS group (c), HJE 0.25 mL/kg + WAS (d), HJE 0.5 mL/kg + WAS (e), PEP 0.25 mL/kg + WAS (f), and PEP 0.5 mL/kg + WAS (g) were observed and representative images of each section were captured at ×100 magnification. Effect of HJE and PEP on TNF-*α* concentration (h), FRAP value (i), and TBARS value (j) on WAS colon. Data were expressed as mean ± SEM (*n* = 6). ^#^*P* < 0.05 versus normal group; ^*∗*^*P* < 0.05 and ^*∗∗∗*^*P* < 0.001 versus WAS group.

The infiltration of inflammatory cells and edema and the depth and area of ulceration on the colonic sections were evaluated by H&E staining. There was no ulceration or infiltration in the normal group (Figure 7(a)). In WAS group (Figure 7(b)), ulceration in muscularis externa, severe inflammatory cell infiltration, edema in the submucosa, and destroyed structures in the mucosa layer were observed. The loperamide-treated group showed severe inflammatory cell infiltration and slight edema only (Figure 7(c)). The low-dose (00.25 mL/kg) HJE-treated group showed slight morphological destruction in the mucus area but did not show inflammatory cells (Figure 7(d)). At high dose (0.5 mL/kg) HJE-treated group revealed morphological changes in the mucosa area and slight edema (Figure 7(e)). Both PEP-treated groups (0.25 and 0.5 mL/kg) showed slight edema and slight mucosal layer destruction (Figures 7(f) and 7(g)). No inflammatory cell infiltration was observed in both HJE- and PEP-treated groups.

### 3.7. Anti-Inflammatory Effect of HEJ and PEP in the Colon of WAS Rats

TNF-*α* is an inflammatory cytokine and is representative of the acute phase of inflammatory reaction. Detection of the TNF-*α* level in the colon by ELISA (Figure 7(h)) showed that the cytokine was increased significantly in the IBS rats compared to the normal rats. It was decreased significantly in rats pretreated for 10 days with loperamide (5 mg/kg), PEP (0.25 and 0.5 mL/kg), and high-dose HJE (0.5 mL/kg) compared to the IBS rats.

### 3.8. Antioxidant Effect of HEJ and PEP in the Colon of WAS Rats

**Figure 8:**
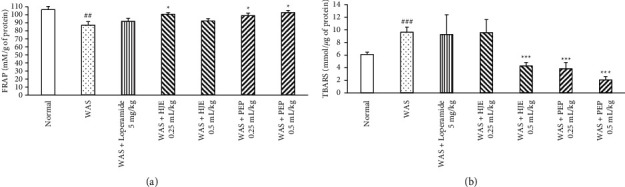
Effect of HJE and PEP in antioxidative stress of WAS colon. Effect of HJE and PEP on FRAP value (a) and TBARS value (b) on WAS colon. The colon homogenates were obtained after 10 days of WAS test and analyzed FRAP and TBARS. Data were expressed as mean ± SEM (*n* = 6). ^###^*P* < 0.001 and ^##^*P* < 0.01 versus normal group; ^*∗*^*P* < 0.05 and ^*∗∗∗*^*P* < 0.001 versus WAS group.

The total antioxidant power of the colon in the WAS group was significantly lower than that in the normal group. 5 mg/kg loperamide did not recover the FRAP level in the colon. At 0.25 mL/kg, HJE significantly recovered the FRAP level diminished by IBS. High-dose HJE (0.5 mL/kg) did not increase the FRAP level. PEP (0.25 and 0.5 mL/kg) group increased the FRAP level significantly when compared to the WAS group (Figure 8(a)).

### 3.9. Antioxidative-Stress Effect of HJE and PEP in the Colon of WAS Rats

As an index of lipid peroxidation, TBARS level was measured to determine oxidative stress (Figure 8(b)). This indicator was significantly higher in the WAS group than in the normal group. 5 mg/kg loperamide did not reduce the TBARS level elevated by IBS. PEP (0.25 and 0.5 mL/kg) and high-dose (0.5 mL/kg) HJE significantly lowered the TBARS level compared to the WAS group, whereas no reduction occurred with low-dose HJE (0.25 mL/kg).

## 4. Discussion

IBS has been considered as a condition arising from dysregulation of the brain-gut axis and classified as a functional GI disorder. Therefore, its symptoms could not be explained by structural or biochemical abnormalities [28]. One of the main characteristics of IBS is visceral hypersensitivity (visceral hyperalgesia). Visceral hyperalgesia can be induced in rodent models by WAS. The present study aimed to evaluate the effects of HJE and PEP on an established model of GI disorders induced by WAS. Methods of GE, GIT, and colonic contractility were applied to investigate the effects of HJE and PEP on the GI motor functions. In addition, to assess the HJE and PEP effects on colonic inflammation in WAS-induced GI disorders, the concentration of Evans blue for colonic permeability and oxidative stress biomarkers were examined.

The chromatographic profile of ethanolic extract of *Humulus japonicus* contains the chemical constituents, luteolin-7-D-glucose derivatives as main components. The active constituents of HJE are luteolin, luteolin-7-D-glucoside, quercetin, and quercitrin. In PEP, the purified polysaccharide was acidic polysaccharide PRP-2. The average molecular weight of PRP-2 was 20332 Da with a polydispersity index of 1.23. The carbohydrate compositions of PRP-2 were D-galactose, L-1-1-fucose, and a small amount of D-rhamnose [29].

In the present study, Wistar rats were used because of their high anxiety level compared to other rat strains [30]. Repeated WAS has been used to build animal models of IBS with visceral hypersensitivity, motility impairment, anxiety, and colonic immune activity [31]. In IBS models, the mean fecal pellet number was increased between days 6 and 10 during WAS session [32]. It was consistent with the present data that showed WAS also increased the mean fecal pellet number. Loperamide was used as a positive control because it possesses antidiarrheal properties as an opiate agonist. In addition, it inhibits the calcium channels and calmodulin and prostaglandin [33]. In the present study, loperamide also reduced the fecal pellet number. Both HJE- and PEP-pretreated rats decreased the fecal pellet number in WAS rats. Therefore, it can be suggested that HJE and PEP could relieve diarrhea in chronic stress-induced GI disorders. The doses of each extract are selected from the pilot study. In the pilot study, the doses ranging from 0.1 to 5 mL/kg were screened. We found that the rats given the doses higher than 0.5 mL/kg produced more fecal pellet output than control ones (data not provided). As a result, the lower doses (0.1, 0.25, and 0.5 mL/kg in logarithmic based doses) of the extracts were used for further experiments.

It was reported that IBS did not alter the GE but increased gastric contraction [34]. GE and gastric contractions do not always correspond. For instance, restraint stress delayed GE but enhanced gastric contractions [35, 36]. One study observed delayed GE in the initial phase (until 24 h) of continuous stress but increased in the late phase (after 24 h) of continuous stress, resulting in decreased levels of adrenalin and noradrenalin. The present results agreed that GE was increased in WAS rats [37]. Quercetin of HJE relaxed the human gastric smooth muscle directly via potassium channels [38]. Galactose, active constituent of PEP, inhibited the GE of young pig initiated by an osmotic effect within the mucosa after absorption [39, 40]. When the WAS rats were pretreated with aqueous HJE and PEP separately, the GE was decreased. These findings suggest that HJE and PEP could be effective for stress-induced GI disorders.

GIT, as a measure of small intestinal motility, was not altered in WAS rats and the HJE and PEP also did not affect that. The relation between GIT and stomach motility remains complex. The stomach and small intestine motility are controlled by intrinsic control mechanisms in the myenteric plexus within the gut wall, which can be modulated by local intrinsic or extrinsic stimuli or by the central nervous system. During the fasting condition, the stomach and small intestine are modulated by a cyclical pattern of contractile activity, which is diminished and replaced by a more random but continuous contractile pattern shortly after a meal [41]. In both GE and GIT experiments, the rats were fasted overnight and were given phenol red 15 min before the tests. At this time, a random and contractile pattern could occur. That might be one of the reasons why WAS-induced GI disorders did not alter the GIT but increased GE.

However, the detailed mechanism of the colonic dysfunction in WAS remains elusive. Colonic contractility was induced by EFS, ACh, and 5-HT in the present research. In the present study, the colonic contractility caused by EFS and ACh was increased in WAS rats. These data were consistent with other findings that a severe nonspecific inflammatory insult to the distal colon of neonatal maternal separation-induced IBS increased the smooth muscle reactivity to ACh [42]. Changing the motor function of the colon in IBS is associated with the cholinergic receptors in the enteric nervous system. Chronic stress rats accelerated the colonic contractility produced by ACh [43]. Luteolin also suppressed the colonic motility mainly acting on L-type calcium channels [44]. Another active constituent of HJE, quercetin, promoted the GI motility in loperamide-induced constipation in the rats by regulating the downstream signaling of the muscarinic acetylcholine receptors [45]. Pretreatment with HJE and PEP reversed the increased colonic contractility induced by EFS and ACh in WAS models. Low-dose HJE (0.25 mL/kg) seemed to be more effective than high-dose HJE (0.5 mL/kg) in inhibiting the EFS-induced colonic contraction. This result could be explained by the antagonistic effect of several bioactive compounds found in the natural plant extract HJE on EFS-induced colonic contraction because colonic smooth muscle cells are simultaneously, directly, and nearly equally stimulated by EFS and cause the release of neurotransmitters including ACh and 5-HT [46]. In addition, there was a high individual variation of SEM. The high-dose tolerance might also be one reason why the low dose seemed to be more effective than the high dose. Therefore, it can be suggested that HJE and PEP acted on the cholinergic system to depress the colonic hypercontractility induced by WAS.

Chronic stress results in the activation of endogenous 5-HT3 receptors responsible for GI functions, and their activation is believed to enhance the release of ACh from parasympathetic nerve terminals [47]. The present study showed that 5-HT induced colonic contractility was increased in the IBS group. The data was consistent with one recent study; the tension due to the ACh-induced colonic smooth muscle contractility was greater in serotonin reuptake transporter-knocked out rats than in wild-type rats [48]. Pretreatment with HJE and PEP inhibited the 5-HT-induced hypercontractility of the colon in the IBS rats. Therefore, it can be concluded that HJE and PEP improved the colonic motor dysfunction in WAS models by targeting the serotonin signaling pathways, in addition to the cholinergic pathways.

It has been demonstrated that WAS stimulates visceral hyperalgesia and colonic hyperpermeability [24]. In recent study, it was shown that abdominal pain in IBS was caused by food antigens-induced allergic immune response through IgE, causing increased intestinal permeability [49]. Stress could also change the intestinal barrier function, by inducing a biphasic increase in the colonic paracellular permeability in mice [50]. Another study showed that dysregulation of claudin-1 protein expression adjusts the p-ERK expression/activity to induce Notch-signaling and dysregulate the colonic epithelial homeostasis, causing inflammatory conditions and hyperplasia [51]. These findings were consistent with the present study where WAS rats showed decreased claudin-1 and OCLN protein expressions. Pretreatment with HJE upregulated the claudin-1 and OCLN expressions. PEP upregulated the OCLN expression in the WAS colon. The present data suggested that HJE and PEP improved the colonic hyperpermeability via upregulating the TJ proteins.

There were no differences in histopathological studies between normal and IBS mice/rats [52]. In recent studies, the stress-induced IBS colon showed complete degeneration of mucus, damaged mucus glands, hyperplasia of the epithelium, and severe inflammatory cell infiltration in the submucosa and the lamina propria [53] and edema [54]. These studies were consistent with the present histological findings that WAS damaged the colonic epithelium and increased the inflammatory cells infiltration and hyperplasia. Loperamide group showed inflammatory cell infiltration in submucosa with no structural changes. This is because loperamide was used as first-line therapy in IBS to stop diarrhea and could lead to constipation [55]. Luteolin and quercetin of HJE reduced the inflammatory cell infiltration, as well as ulceration in ulcerative colitis rats [56, 57]. According to several studies, polysaccharides have a lot of beneficial effects on intestinal epithelial barrier function [58]. Administration of polysaccharide-based nutrition formula from *Crassostrea hongkongensis* to the 5-fluorouracil-treated rats reduced the intestinal mucosal ulceration and increased the mucosa thickness [59]. Fucose, active constituent of PEP, restored the bile acids and gut microbiota and improved the chronic colitis induced by dextran sulfate sodium in mice [60]. Consequently, 10-day pretreatment with HJE and PEP showed that the muscularis externa and mucosa layers were repaired. These findings suggested that HJE and PEP could effectively promote the function of the intestinal epithelial barrier in WAS rats.

Increased intestinal permeability in IBS leads to free passage of antigens (bacteria or foods) into the cells. The leaky intestine can upregulate the mast cells through T-helper-2 cell pathway, producing the inflammatory cytokines, histamine, and serotonin [61]. IBS promoted gut hypersensitivity and abdominal pain with a low-grade mucosal inflammation accessed by increased cytokine levels and immune activation [62]. It was reported that the expressions of TNF-*α* and NF-*κ*Bp65 were increased in IBS mice [63]. TNF-*α* concentration was also increased in colon samples after 5-day exposure to immobilization stress [64]. These findings were consistent with the present experiment that TNF-*α* concentration was increased in WAS rats. Subsequently, the pretreatment of HJE and PEP showed a decreased concentration of TNF-*α* in IBS rats. HJE has a potential protective effect in acute liver injury by decreasing the levels of TNF-*α* [65]. These findings suggested that HJE and PEP improved the colonic hyperpermeability and inflammation in IBS rat models by reducing the proinflammatory cytokines.

**Figure 9:**
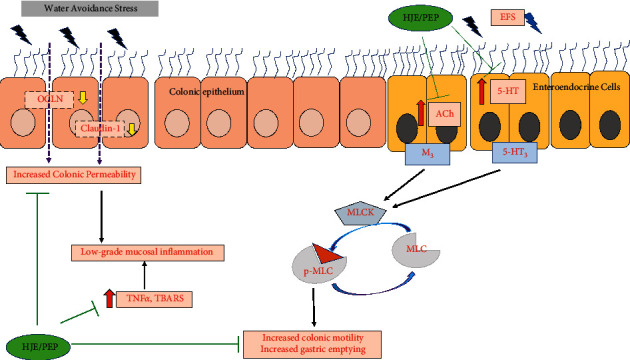
Proposed mechanisms of HJE and PEP on WAS-induced gastrointestinal disorders.

In addition, quantifying the oxidative stress is valuable for determining the severity of inflammation [66]. Stress-induced IBS has been shown to increase the TBARS level and decrease the FRAP levels in the colon [26]. These findings were consistent with the present experiments where WAS increased the TBARS levels, while FRAP value was decreased. The methanol extract of HJE increased glutathione levels and decreased lipid peroxidation in 6-hydroxydopamine-lesioned mice [67]. In one study, luteolin reduced the levels of TBARS and hydroxy radicals against the azoxymethane-induced colon cancer tissues in mice [68]. PEP-treated rats attenuated the increase of myeloperoxidase activity and TBARS level in water immersion stress rats [17]. In the present study, pretreatment with HJE and PEP recovered the TBARS and FRAP values in WAS rats. Therefore, HJE and PEP were suggested to exert therapeutic effects on oxidative stress-associated factors. To sum up, the proposed mechanisms of HJE and PEP on WAS-induced gastrointestinal disorders are provided in Figure 9.

Chronic WAS causes the GI symptoms closely related to IBS in rat models. WAS induces the low-grade mucosal inflammation in colon, which leads to the release of inflammatory cytokines like TNF-*α*, and elevation of lipid peroxidation, together with a decrease in the antioxidant FRAP value in the colon. This mucosal inflammation can further cause increased colonic permeability in the epithelium by downregulating the TJ proteins (OCLN and claudin-1). In addition, the colonic contractile responses against the EFS, Ach, and 5-HT were elevated by WAS. Pretreatment with HJE and PEP for 10 days exerts antioxidant effect by lowering the TNF-*α* and TBARS levels and increasing FRAP value. As a result, PEP and HJE recover the colonic hyperpermeability caused by WAS via upregulating the expressions of claudin-1 and occludin. Moreover, HJE and PEP blocked increased gastric emptying and the colonic contractile responses due to the EFS, ACh, and 5-HT in IBS rats. 5-HT3: serotonin receptor 3, M3: muscarinic receptor 3, MLCK: myosin light chain kinase, MLC: myosin light chain, p-MLC: phosphorylated form of myosin light chain.

## 5. Conclusion

The present study strongly suggests that HJE and PEP decreased the defecation, GE, and colonic contractility in WAS rat models by inhibiting the cholinergic and serotonergic signaling pathways. In addition, HJE and PEP improved the colonic permeability and upregulated the TJ proteins (OCLN and claudin-1) with the degradation of inflammatory cytokines (TNF-*α*) and oxidative stress, as well as elevation of antioxidant capability in WAS rats. WAS can also induce visceral hyperalgesia and GI disorders are hallmark symptoms of IBS. Therefore, it is proposed that HJE and PEP might be potential therapeutic agents for IBS.

## Data Availability

The data used to support the findings of this study are available from the corresponding author upon request.

## Abbreviations

5-HT: 5-Hydroxytryptamine or serotonin
ACh: Acetylcholine
ANOVA: Analysis of variance
BCA assay: Bicinchoninic acid assay
EFS: Electrical field stimulation
ELISA: Enzyme-linked immunosorbent assay
FRAP: Ferric reducing antioxidant power
GE: Gastric emptying
GI: Gastrointestinal
GIT: Gastrointestinal transit
H&E: Hematoxylin and eosin
HJE: *Humulus japonicus* extract
IBS: Irritable bowel syndrome
IL-1*β*: Interleukin-1*β*
MDA: Malondialdehyde
MGC: Mean geometric center
OCLN: Occludin
PBS: Phosphate-buffered saline
PEP: Polysaccharide-rich extract of *Phragmites rhizoma*
RIPA: Radioimmunoprecipitation assay buffer
SDS-PAGE: Sodium dodecyl sulfate-polyacrylamide gel electrolysis
TBARS: Thiobarbituric acid reactive substances
TJ: Tight junction
TNF-*α*: Tumor necrotic factor-*α*
TPTZ: 2,3,5-triphenyltetrazolium chloride
WAS: Water avoidance stress.
